# AI-Assisted OCT Clinical Phenotypes of Diabetic Macular Edema: A Large Cohort Clustering Study

**DOI:** 10.3390/jcm14227893

**Published:** 2025-11-07

**Authors:** Edoardo Midena, Marco Lupidi, Lisa Toto, Giuseppe Covello, Daniele Veritti, Elisabetta Pilotto, Maria Vittoria Cicinelli, Rosangela Lattanzio, Michele Figus, Giulia Midena, Luca Danieli, Enrico Borrelli, Michele Reibaldi, Daniele Tognetto, Leandro Inferrera, Simone Donati, Settimio Rossi, Paolo Melillo, Paolo Lanzetta, Valentina Sarao, Giulia Gregori, Carlo Cagini, Chiara Maria Eandi, Adriano Carnevali, Vincenzo Scorcia, Emilia Maggio, Grazia Pertile, Ciro Costagliola, Gilda Cennamo, Paolo Mora, Roberto Dell’Omo, Marzia Affatato, Marzia Passamonti, Mariacristina Parravano, Nicola Vito Lassandro, Marco Nassisi, Francesco Viola, Niccolò Castellino, Francesco Cappellani, Giuseppe Giannaccare, Francesco Boscia, Maria Oliva Grassi, Donatella Musetti, Valentina Folegani, Alessandro Invernizzi, Luca Rossetti, Tommaso Bacci, Federico Ricci, Marco Lombardo, Mary Romano, Nicola Valsecchi, Michele Coppola, Fabiano Cavarzeran, Luisa Frizziero

**Affiliations:** 1 Department of Ophthalmology, University of Padova, 35100 Padova, Italy; 2 IRCCS—Fondazione Bietti, 00184 Rome, Italy; 3 Department of Experimental and Clinical Medicine, Polytechnic University of Marche, 60121 Ancona, Italy; 4 Ophthalmology Clinic, Department of Medicine and Science of Ageing, “G. d’Annunzio” University Chieti-Pescara, 66100 Chieti, Italy; 5 Ophthalmology, Department of Surgical, Medical, Molecular Pathology and Clinical Care Medicine, University of Pisa, 56126 Pisa, Italy; 6 Department of Medicine-Ophthalmology, University of Udine, 33100 Udine, Italy; 7 Istituto Europeo Di Microchirurgia Oculare—IEMO, 33100 Udine, Italy; 8 School of Medicine, Vita-Salute San Raffaele University, 20132 Milan, Italy; 9 Department of Ophthalmology, IRCCS San Raffaele Scientific Institute, 20132 Milan, Italy; 10 Department of Ophthalmology, City of Health and Science Hospital, University of Turin, 10126 Turin, Italy; 11 Eye Clinic, Department of Medical, Surgical Sciences and Health, University of Trieste, 34128 Trieste, Italy; 12 Ophthalmology Unit, Circolo Hospital, Department of Medicine and Surgery, University of Insubria, 21100 Varese, Italy; 13 Multidisciplinary Department of Medical, Surgical and Dental Sciences, Eye Clinic, University of Campania “Luigi Vanvitelli”, 81131 Naples, Italy; 14 Department of Medicine and Surgery, University of Perugia, 06132 Perugia, Italy; 15 Department of Ophthalmology, University of Lausanne, Jules-Gonin Eye Hospital, Fondation Asile des Aveugles, 1004 Lausanne, Switzerland; 16 Department of Surgical Sciences, University of Torino, 10124 Torino, Italy; 17 Department of Ophthalmogy, University Magna Graecia of Catanzaro, Azienda Ospedaliera-Universitaria R. Dulbecco, 88100 Catanzaro, Italy; 18 Ophthalmology Unit, IRCCS Sacro Cuore Don Calabria Hospital Verona, 37024 Verona, Italy; 19 Eye Clinic, Department of Neurosciences, Reproductive Sciences and Dentistry, University of Naples Federico II, 80131 Naples, Italy; 20 Ophthalmology Unit, University Hospital of Parma, 43126 Parma, Italy; 21 Department of Medicine and Health Sciences “Vincenzo Tiberio”, University of Molise, 86100 Campobasso, Italy; 22 Faculty of Medicine, UniCamillus-Saint Camillus International University of Health Sciences, 00131 Rome, Italy; 23 Fondazione IRCCS Cà Granda Ospedale Maggiore Policlinico, 20122 Milan, Italy; 24 Department of Clinical Sciences and Community Health, University of Milan, 20122 Milan, Italy; 25 Department of Ophthalmology, University of Catania, 95123 Catania, Italy; 26 Department of Medicine and surgery, University of Enna “Kore”, 94100 Enna, Italy; 27 Eye Clinic, Department of Surgical Sciences, University of Cagliari, 09124 Cagliari, Italy; 28 Department of Translational Biomedicine Neuroscience, University of Bari “Aldo Moro”, 70121 Bari, Italy; 29 Clinica Oculistica DiNOGMI, Università di Genova, Ospedale Policlinico San Martino IRCCS, 16132 Genova, Italy; 30 Eye Clinic, Department of Biomedical and Clinical Sciences, Ospedale Luigi Sacco, University of Milan, 20157 Milan, Italy; 31 Eye Clinic, ASST Santi Paolo e Carlo—San Paolo Hospital, University of Milan, 20142 Milan, Italy; 32 Ophthalmology Unit, Department of Medicine, Surgery and Neuroscience, Siena University Hospital, 53100 Siena, Italy; 33 Retinal Diseases Unit-PPTV, Department of Experimental Medicine, University of Rome Tor Vergata, 00133 Roma, Italy; 34 Department of Ophthalmology, IRCCS Humanitas Research Hospital, Rozzano, 20089 Milan, Italy; 35 Multidisciplinary Department of Medical, Surgical and Dental Sciences University of Campania Luigi Vanvitelli, 80138 Neaples, Italy; 36 Ophthalmology Unit, IRCCS Azienda Ospedaliera-Universitaria di Bologna, 40138 Bologna, Italy; 37 IRCCS-San Gerardo, 20900 Monza, Italy

**Keywords:** diabetic macular edema, biomarkers, optical coherence tomography, intraretinal fluid, subretinal fluid, hyperreflective retinal foci, external limiting membrane, ellipsoid zone, clustering analysis, clinical phenotypes

## Abstract

**Purpose:** To characterize, using clustering analysis, the OCT morphological and clinical phenotypes of diabetic macular edema (DME) in a very large population (>2000 DME eyes) using standardized and validated OCT-based biomarkers. **Methods:** A cross-sectional study was conducted on OCT scans collected from 2355 eyes of 1688 patients with DME and performed during real-world clinical practice. OCT scans were automatically analyzed by a software able to automatically quantify OCT key biomarkers: intraretinal fluid (IRF), subretinal fluid (SRF), hyperreflective retinal foci (I-HRF), and external limiting membrane (ELM) and ellipsoid zone (EZ) interruption. Clustering analysis was performed using the above-mentioned biomarkers, including the distribution of IRF across the three ETDRS rings. **Results:** The overall population was predominantly composed of type 2 diabetes patients (89%), with a mean diabetes duration of 15.6 ± 10.7 years and mean best corrected visual acuity (BCVA) of 63 ± 18 ETDRS letters. Multivariate clustering identified four morphological phenotypes with distinct patterns of fluid distribution associated with different I-HRF counts, SRF volume, and percentages of ELM/EZ integrity (*p* < 0.0001). **Conclusions:** This large OCT analysis identified distinct morphological subtypes of DME, confirming the clinical relevance of key imaging biomarkers. The distribution and severity of DME features differ among clusters, supporting the importance of OCT-based phenotyping in tailoring treatment strategies and understanding disease evolution.

## 1. Introduction

Diabetic macular edema (DME) is a leading cause of visual impairment among working-age adults with diabetes, representing a significant public health burden worldwide [1]. Both the number of cases and the prevalence of diabetes have been steadily increasing over the past few decades, and diabetes mellitus (DM) is considered a global epidemic of the 21st century with about 589 million adults (20–79 years) affected worldwide [2]. In Italy, diabetes affects about 5 million people, with an age-standardized prevalence of about 7.7%, a parallel growing incidence of diabetic retinopathy (DR) and DME, and important implications for the healthcare system [3]. The clinical presentation, progression, and response to treatment of DME can vary widely depending on different factors [4]. For several years, grid and focal laser photocoagulation were considered the standard of care for this eye disease [5]. With the advent and constantly increasing number of therapeutic options, the scenario has changed and the advancements in retinal diagnostic imaging techniques have shown the possibility to detect DME features possibly related to specific pathophysiological mechanisms, prognostic evidences, and therapy responses [6,7]. Despite advances in diagnostic imaging and therapeutic approaches, real-world large data on the characteristics and management of DME remain limited. Understanding the clinical features of DME (clinical phenotypes) in this context is crucial for optimizing patient care and resource allocation. The aim of this paper is to report a representation of the clinical and imaging features characterizing patients with DME, using a validated and standardized method of AI-assisted optical coherence tomography (OCT) image analysis.

## 2. Materials and Methods

This paper reports the results of the analysis performed on the major validated OCT biomarkers of DME, obtained from OCT scans collected in the largest population of DME eyes reported in the literature.

OCT scans acquired using the Spectralis platform (Heidelberg Engineering, Heidelberg, Germany) and according to standardized criteria were analyzed: a 6 × 6 mm volumetric map of 49 scans acquired in High Speed (HS) modality with >12 ART and a quality index > 28, and a linear scan passing through the fovea acquired in High Resolution (HR) mode with >90 ART and a quality index > 30. All scans were centered onto the fovea. Only scans collected from eyes affected by DME were included. Exclusion criteria were any sign of chorioretinal diseases other than diabetic macular edema (e.g., drusen).

Each scan was separately analyzed by masked examiners with the same software for automatic (AI) quantification, as previously described [8]. For each volumetric scan the total volume of the intraretinal (IRF) and subretinal fluid (SRF) in the 6 × 6 map were reported as well as the percentage of distribution of the IRF in the Early Treatment Diabetic Retinopathy Study (ETDRS) macular rings of 1, 3, and 6 mm. The percentage of interruption of the external limiting membrane (ELM) and ellipsoid zone (EZ) were analyzed in the central mm of the central scan. Moreover, for each eye the number of inflammatory hyperreflective retinal foci (I-HRF) was automatically calculated and reported in the central 3 mm of the linear HR scan passing through the fovea.

Central subfield thickness (CST) and best corrected visual acuity (BCVA) were also reported for each case. Finally, for each case the following data were collected: previously treated or untreated DME eyes and, for previously treated, the time from first treatment; duration and type of diabetes (1 or 2); proliferative or non-proliferative DR; and the presence of epiretinal membrane (ERM) as clinically evaluated according to the definition provided by Govetto et al. [9].

Local examiners verified the inclusion and exclusion criteria as well as the quality of the images of all data collected, after anonymization. Specifically, all OCT scans were subjected to quality control before inclusion. This process included the following steps: verification of proper foveal centering; assessment of image ART (>12 for volumetric map scan and >90 for HR linear scan); and assessment of quality index (>28 for volumetric map scan and >30 for HR linear scan). To ensure the correctness of the process, values for the quality index of the volumetric map scan were collected from all centers.

### Statistical Analysis

Study parameters were summarized according to the usual methods provided by descriptive statistics: quantitative parameters were summarized as average ± standard deviation (SD) and categorical parameters were described as absolute and relative (percentage) frequencies.

Model-based clustering (Mclust R package, version 6.1.1 [10]) was performed to identify latent subgroups within the data. The following parameters were selected: IRF volume, IRF % distribution, SRF volume, I-HRF count, and ELM and EZ percentage of interruption. The IRF distribution in the 3–6 mm ETDRS ring was excluded from this analysis because it is highly correlated—according to Pearson’s correlation coefficient—with central and 1–3 mm measures. All such parameters were square-root transformed to approximate normality as they presented skewed distributions. A multivariable Gaussian finite mixture model was applied to the scaled dataset using the VEV structure (ellipsoidal clusters with equal shape). Optimal clustering was based on the Bayesian information criterion. The presence of ERM was not included in this analysis due to the lack of automated quantitative assessment, as this parameter was not evaluated by the software. Therefore, it was considered only to assess potential differences among clusters, similarly to the other clinical parameters. The normality of data distribution was tested using the Kolmogorov–Smirnov test. Since some patients contributed both eyes to the analysis, for data which did not follow a normal distribution, differences among clusters were analyzed using a repeated-measures ANOVA applied to the ranks of the measurements across the entire sample, followed by the Tukey–Kramer post hoc test for pairwise comparisons

For all statistical analyses SAS^®^ v. 9.14 (SAS Institute, Cary, NC, USA) and R (R Core Team, Vienna, Austria, version 4.3.1) software were used. Statistical tests were interpreted as significant if *p* < 0.05.

## 3. Results

### 3.1. Population

Data from 2376 eyes were collected. Twenty-one eyes, corresponding to 0.9% of the population, were excluded because of missing data, i.e., previous treatment. After exclusion, data from 2355 eyes of 1688 patients were analyzed. Mean CST was 386 ± 123 µm and mean BCVA was 63 ± 18 ETDRS score.

The mean age of the patients whose scans were analyzed was 67.7 ± 10.7 years and the majority of patients were affected by type 2 diabetes (1507, 89%). Mean diabetes duration in the whole population was 15.7 ± 10.7 years, with a greater duration in type 1 patients (25.8 ± 16.0 years) compared to type 2 patients (14.5 ± 9.1 years). A total of 1677 eyes (71.2%) were affected by non-proliferative DR (NPDR), while 678 (28.8%) eyes by proliferative DR. Mean DME duration (time from first treatment) was 2.9 ± 3.0 years (Table 1).

From a morphological point of view, the presence of ERM was detected in 736 eyes, corresponding to 31.3% of the whole population. The mean IRF volume was 0.799 ± 1.170 mm^3^, with a mean of 18 ± 21% of fluid located in the center 1 mm circle, 37 ± 20% in the middle ETDRS ring, and 45 ± 29% in the peripheral ETDRS ring. SRF was identified in 301 eyes (12.8%) with a mean volume of 0.047 ± 0.086 µm^3^, ELM interruption in 516 eyes (21.9%), and EZ interruption in 886 eyes (37.6%). The mean number of I-HRF detected in the population was 81.4 ± 28.6 (Table 1).

### 3.2. Clustering

The multivariate clustering analysis identified a four clusters model as the one with the lowest Bayesian information criterion (BIC, −27,431.17). The four clusters’ characteristics are reported in Table 2. The normality test (Kolmogorov–Smirnov test) showed a non-normal distribution of all the clustered variables (*p* < 0.0100). A significantly different distribution was found in terms of IRF volume and distribution, SRF volume, ELM and EZ percentage of interruption (*p* < 0.0001), and I-HRF number among the four clusters (*p* = 0.0003). (Table 2)

The four clusters also differed for age (67.4 ± 111, 66.0 ± 10.6, 69.2 ± 9.5, 67.7 ± 10.6 for cluster 1, 2, 3, 4, respectively, *p* < 0.0001), CST (349.8 ± 74.0, 356.4 ± 99.6, 396.2 ± 127.7, 445.9 ± 152.2 for cluster 1, 2, 3, 4, respectively, *p* < 0.0001), BCVA (69.4 ± 14.1, 67.2 ± 15.8, 59.6 ± 17.4, 55.6 ± 19.0 for cluster 1, 2, 3, 4, respectively, *p* < 0.0001), DME duration (2.9 ± 3.3, 2.6 ± 2.8, 3.4 ± 3.3, 2.8 ± 2.8 for cluster 1, 2, 3, 4, respectively, *p* = 0.039), presence of PDR (166 (25.6%), 178 (27.4%), 141 (33.7%), 193 (30.2%) eyes in cluster 1, 2, 3, 4, respectively, *p* = 0.0270), and presence of ERM (169 (26.1%), 203 (31.3%), 140 (33.4%), 224 (35.1%) eyes with ERM in cluster 1, 2, 3, 4, respectively, *p* = 0.0044). No statistically significant difference was found among clusters, considering type and duration of diabetes.

## 4. Discussion

Diabetic retinopathy (DR) involves about 30% of the population with diabetes and DME is the major cause of vision loss associated with DR. It is characterized by the accumulation of fluid in the macula due to alteration of the homeostasis of the retinal neuro-glial-vascular unit associated with retinal thickening, leading to some characteristic symptoms, including visual blurring and metamorphopsia, causing a relevant limitation of daily life activities [11]. DME has a multifactorial origin, involving not only elevated VEGF expression but also contributions from inflammation and neurovascular unit impairment [4,12,13]. Anti-VEGF therapies primarily target one aspect of this complex condition, which may account for why a significant percentage of patients continue to experience persistent edema and up to 40% show limited visual improvement, despite regular treatment [14,15]. In these cases, an early switch may lead to better outcomes as compared to a late switch, suggesting the importance of identifying patients possibly responding better to one therapy rather than another [16]. Furthermore, challenges such as the disconnection between anatomical and functional outcomes, the high financial burden of treatment, and variability in individual responses complicate patient management. There is a clear need to enhance the ability to predict treatment response in DME, enabling clinicians and patients to make more informed decisions when choosing a therapeutic strategy [17]. Numerous imaging parameters are continuously being proposed to better characterize DME, aiming to improve diagnosis, prognostication, and treatment guidance. These include features such as retinal fluids, hyperreflective retinal foci, external retinal integrity, disorganization of the retinal inner layers (DRIL), and quantitative metrics derived from OCT angiography [7,18,19]. OCT is considered the gold standard for the diagnosis and follow-up of DME due to its high-resolution and non-invasive imaging capabilities, since it provides detailed cross-sectional images of the retina [18]. Several studies have attempted to classify DME into distinct morphological subtypes based on OCT features—such as cystoid, diffuse, and serous retinal detachment patterns—with the goal of better understanding disease mechanisms and tailoring treatment approaches. Although these classifications offer useful insights into the structural variability of DME, they have not been translated into meaningful applications in either clinical practice or research. To date, no universally accepted morphological system has demonstrated clear prognostic value or led to changes in therapeutic decision-making. As a result, these subtype-based approaches remain largely descriptive and have limited impact on guiding patient management or predicting treatment outcomes.

Following earlier attempts at classifying DME into broad morphological patterns, research has progressively identified specific structural biomarkers that have gained relevance and are now increasingly incorporated into clinical practice and research. Parameters such as IRF, SRF, I-HRF, ELM, and EZ have shown associations with visual prognosis and treatment response. These features, detectable and quantifiable on OCT, have enhanced the ability of clinicians to assess disease severity, monitor progression, and make more informed therapeutic decisions [7,8,18].

AI is increasingly showing its relevant role in the automatic analysis of data obtained from ocular imaging in several diseases [20]. By automating the detection and quantification of key structural features, AI enhances consistency, reduces observer variability, and allows for more objective, time-saving and reproducible assessments. These technologies are bridging the gap between research findings and clinical applicability, making advanced biomarker analysis more accessible in routine practice. As AI tools continue to evolve and integrate into imaging platforms, they are expected to play an increasing role in personalized treatment planning and real-time decision-making for patients with DME [20].

The application of an AI software (version 1.0) specifically developed and validated for DME allowed the cross-sectional analysis of OCT scans collected from a huge number of eyes affected by DME during real-world clinical practice. The possibility to quantify in a repeatable way major OCT biomarkers of DME, allowed the application of a clustering analysis, based on IRF and SRF volumes, IRF distribution and quantification of I-HRF, and ELM and EZ interruption. No other morphological parameters were included in the analysis since automatic quantification was not available for other OCT features. In particular, DRIL was not included, despite its increasingly recognized role in visual prognosis in DME. In fact, at present the quantification of DRIL is still not standardized and no available software has been validated for its measurement [21]. Its inclusion should be prioritized in future multicenter imaging studies using standardized acquisition protocols.

The clustering analysis conducted on a large real-world cohort of eyes with DME allowed the identification of four distinct morphological phenotypes: cluster 1 (27.5% of the population) was characterized by low IRF, mainly located in the central macula (34% within the 0–1 mm ring); absence of SRF; and intact ELM/EZ. The mean number of I-HRF was the lowest across clusters (77.1 ± 24.4), significantly inferior to cluster 3 and 4 (*p* = 0.0084 and *p* = 0.0004, respectively). Such features suggest an early or less active form of DME with largely preserved retinal architecture and limited neuroinflammatory activity, despite the duration of diabetes and DME being overall comparable to that of other clusters. Functionally, this phenotype may correspond to cases with relatively good visual potential, where the neurovascular unit and outer retinal structures remain functionally competent despite disease duration comparable to that of other clusters. Therefore, we can consider this cluster a mild, localized DME with preserved retinal integrity. In cluster 2 and 3 the IRF volume was significantly higher than cluster 1 (*p* < 0.0001) and similar to each other (*p* = 0.7610) with a higher standard deviation in cluster 3. However, in cluster 2, the IRF distribution in the ETDRS rings was more peripheral (68.1% within the 3–6 mm ring) compared to cluster 3 (43.0%, *p* < 0.0001 vs. cluster 2), SRF and ELM disruption were virtually absent, and EZ alterations were minimal (mean 0.035%). This configuration likely represents an intermediate DME phenotype, characterized by perifoveal fluid accumulation and preserved outer retinal layers. It might reflect a localized microvascular dysfunction with limited neuroretinal damage, potentially preceding more central or diffuse involvement. Such cases could be more responsive to early therapeutic intervention.

Cluster 3 exhibited an IRF volume similar to cluster 2 (0.847 mm^3^) but with a predominantly central distribution (22.1% within the 0–1 mm ring, *p* < 0.0001 vs. cluster 2). It showed a higher percentage of EZ disruption (29.5%), occasional SRF, and a significantly greater number of I-HRF (84.8 ± 32.2, *p* = 0.0002 vs. cluster 1). The co-occurrence of central fluid accumulation, EZ damage, and increased I-HRF suggests a more advanced photoreceptor and Müller cell dysfunction, possibly reflecting enhanced inflammatory activity and oxidative stress within the central macula. This phenotype may require a more prompt and aggressive treatment to preserve visual function.

Cluster 4 (27.1%) was characterized by the highest IRF volume involving the entire macula, the presence of SRF, and extensive ELM (32.2%) and EZ (41.2%) interruption. It also showed the highest mean number of I-HRF (84.2 ± 29.1). The IRF distribution was generalized (51.3% within the 3–6 mm ring), indicating diffuse retinal edema and global disruption of retinal homeostasis. These eyes likely represent advanced stages of DME, characterized by breakdown of the outer blood–retinal barrier, significant neuroinflammatory activity, and widespread photoreceptor disorganization. Functionally, such morphology is expected to correspond to marked visual impairment and unpredictable treatment responsiveness.

In this population clinical data confirmed the higher prevalence of type 2 diabetes with a longer duration of the disease in type 1 patients. However, type and duration of diabetes did not statistically differ among clusters. This may be due to the wide range of DME eyes involved, as well as to the intrinsic complexity of the disease, where the presence of local and individual factors interacts with systemic ones leading to the different clinical manifestations of DME. The lack of control for the confounding factor of treatment in our dataset may have influenced or masked possible associations between diabetes characteristics and DME morphology; therefore, our findings do not exclude a potential relationship but rather suggest that other local and individual factors may contribute to the heterogeneous morphological manifestations of DME. Future studies are needed to clarify this relationship.

Substantial inter-individual variability in the pathways and severity of neurovascular unit dysfunction has been suggested, even among patients with similar diabetic retinopathy grades [22]. Different mechanisms may contribute to the heterogeneity of morphological changes involving the diabetic retina. Multimodal imaging studies show that neurodegeneration, microvascular damage, ischemia, exudation, and neuroinflammation may variably manifest, with different patterns not always correlated directly with retinopathy severity or duration of diabetes [22,23]. Some data reported in this study showed high standard deviations reflecting the intrinsic heterogeneity of the DME population and the wide clinical variability in disease severity and morphological presentation, particularly regarding structural features. This variability is consistent with the large and representative multicenter population included in the study, which mirrors the diversity typically encountered in real-world clinical settings. Moreover, in clusters 2 and 3 a similar volume of fluid seems to distribute differently, with different percentages of ELM and EZ interruption and I-HRF count (*p* < 0.0001). I-HRF tends to increase in the cluster, showing a more central IRF concentration, as SRF does, which also grows in the fourth cluster, characterized by a more generalized disruption of the overall retinal morphology leading to an increased functional impairment. The distribution of these morphological features may help in identifying patients with a greater alteration of the retinal homeostasis and of the neuroinflammatory mechanisms regulated by the neurovascular unit [24].

The interruption of the ELM is generally less extensive than that of the EZ, suggesting that the EZ is more susceptible to damage. As previously reported, photoreceptors may break down earlier, but they have also the potential to regenerate [25]. The ELM is formed by the junctions among photoreceptors and Müller cells, which provide morpho-functional support to the entire retinal structure. It may show signs of disruption at a later stage, remaining as a point of support for the subsequent restoration of photoreceptors, if possible [26,27].

The prevalence of ERM significantly differed among the four clusters, particularly considering cluster 1 compared to clusters 3 (*p* = 0.0583, borderline) and 4 (*p* = 0.0044). Although ERM was not included in the clustering analysis due to the lack of an automated quantitative assessment, its prevalence confirmed its relevance in the morphological changes involving DME eyes and its distribution across clusters showed a trend towards a higher frequency in eyes with more advanced structural disorganization. This finding may reflect the progressive involvement of the vitreoretinal interface as DME severity increases. However, given the cross-sectional design and the qualitative assessment of ERM, these results should be interpreted with caution, and further studies with fully automated quantitative evaluation are warranted to clarify the role of ERM in the morphological spectrum of DME.

The major strengths of this research are the number of study eyes and the clustering analysis. One limitation of this report may be the use of just OCT imaging modality, however OCT is at present the gold standard for the characterization of DME and its peculiar features in terms of non-invasiveness, cross-sectional visualization, and quantification makes it the only technique allowing a large-scale imaging study. Another limitation may be the absence of a central reading center to analyze all images. However, this limitation is overcome by the fact that all the images were automatically analyzed by the same software in each center, allowing them to maintain the confidentiality of the images in the center of origin.

This study involves both treated and untreated eyes, reflecting a cross-sectional, real-world analysis of the behavior of major OCT biomarkers of DME to identify morphological and clinical phenotypes. Detailed information on treatment type, duration, or intensity was not included in this analysis, since a stratified analysis comparing therapy types or treatment intensity across the identified clusters was beyond the scope of this work. Therefore, the observed phenotypic differences may partly reflect treatment-induced variations rather than purely natural disease subtypes. This limitation underscores the need for future longitudinal studies integrating comprehensive treatment data to analyze the modifications of these phenotypes according to the natural history of the disease and secondary to treatment. Future research should also explore how these phenotypes evolve over time, both according to the natural history of DME and in response to different therapeutic strategies.

## 5. Conclusions

This analysis, including the largest reported population of eyes affected by DME, demonstrated that the use of artificial intelligence-based software applied to high-quality OCT imaging enables the observation of distinct morphological patterns based on quantitative structural biomarkers.

Clustering analysis revealed significant heterogeneity in DME presentation. The distribution of intraretinal and subretinal fluid, the number of retinal hyperreflective foci, and the integrity of the ELM and EZ emerged as key structural parameters that may reflect different degrees of neurovascular unit dysfunction and variable susceptibility to damage. The distribution of fluid seems to be differently associated with other structural and neuroinflammatory biomarkers which also characterized eyes with increased functional impairment.

Overall, these findings highlight how the integration of OCT imaging with automated analysis can improve our understanding of DME morphological variability and support more personalized disease management. This was a cross-sectional and exploratory analysis; the results point to the need for longitudinal studies to investigate how DME phenotypes evolve over time and respond to various therapeutic approaches. Ultimately, such evidence could contribute to more targeted treatment choices and better healthcare resource allocation.

## Figures and Tables

**Table 1:** Clinical and morphological characteristics of the whole population.

Parameter	
Eyes, n	2355
Patients, n	1688
Age, years, mean ± SD	67.7 ± 10.7
Type of diabetes, n (%)	
1	181 (10.7)
2	1057 (89.3)
Diabetes duration, years, mean ± SD	15.7 ± 10.7
1	25.8 ± 16.0
2	14.5 ± 9.2
DR grade, n (%)	
PDR	678 (28.8)
NPDR	1677 (71.2)
Previous treatment, n (%)	
No	404 (17.2)
Yes	1951 (82.8)
DME duration, years, mean ± SD	2.9 ± 3.0
ERM, n (%)	
No	1619 (68.8)
Yes	736 (31.3)
IRF, mm^3^, mean ± SD	0.799 ± 1.170
IRF distribution, %, mean ± SD	
0–1	18.0 ± 20.7
1–3	36.8 ± 19.6
3–6	45.3 ± 29.2
SRF, mm^3^, mean ± SD	0.047 ± 0.086
ELM, %, mean ± SD	39.9 ± 33.9
EZ, %, mean ± SD	43.7 ± 36.2
I-HRF, n, mean ± SD	81.4 ± 28.6
Q index, mean ± SD	30.9 ± 5.3
CST, µm, mean ± SD	386 ± 123
BCVA, ETDRS score, mean ± SD	63 ± 18

SD: standard deviation; DR: diabetic retinopathy; PDR: proliferative DR; NPDR: non-proliferative DR; DME: diabetic macular edema; ERM: epiretinal membrane; IRF: intraretinal fluid; SRF: subretinal fluid (n = 301, 12.8%); ELM: external limiting membrane (n = 516, 21.9%); EZ: ellipsoid zone (n = 886, 37.6%); I-HRF: inflammatory hyperreflective retinal foci; CST: central subfield thickness; BCVA: best corrected visual acuity; ETDRS: early treatment diabetic research study.

**Table 2:** Multivariate clustering analysis in the whole population.

Parameter		Cluster 1	Cluster 2	Cluster 3	Cluster 4	*p*-Value
Eyes, n (%)		648 (27.5)	649 (27.6)	419 (17.8)	639 (27.1)	
IRF, mm^3^	mean	0.155	0.717	0.847	1.504	<0.0001
	SD	0.147	0.707	1.157	1.649	
	median	0.106	0.559	0.421	0.884	
	IQR	0.182	0.825	0.986	1.837	
IRF distribution, %						
0–1	mean	34.0	5.6	22.1	11.5	<0.0001
	SD	23.2	5.7	23.5	12.9	
	median	30	4	13	7	
	IQR	34	9	22	13	
1–3	mean	48.0	26.3	34.9	37.2	<0.0001
	SD	19.4	15.5	19.3	17.5	
	median	49	26	36	36	
	IQR	26	23	27	25	
3–6	mean	18.0	68.1	43.0	51.3	<0.0001
	SD	16.6	19.5	28.3	25.2	
	median	13	69	44	56	
	IQR	30	29	45	38	
SRF, mm^3^	mean	0.00	0.00	0.00	0.022	<0.0001
	SD	0.00	0.00	0.001	0.063	
	median	0	0	0	0	
	IQR	0	0	0	0.010	
ELM, %	mean	0.00	0.00	0.04	32.2	<0.0001
	SD	0.00	0.00	0.22	34.3	
	median	0	0	0	16	
	IQR	0	0	0	63	
EZ, %	mean	0.00	0.035	29.5	41.2	<0.0001
	SD	0.00	0.230	31.4	39.3	
	median	0	0	17	29	
	IQR	0	0	38	82	
I-HRF, n	mean	77.1	80.7	84.8	84.2	0.0003
	SD	24.4	29.0	32.2	29.1	
	median	76	77	81	82	
	IQR	30	35	37	37	

SD: standard deviation; IQR: interquartile range; IRF: intraretinal fluid; SRF: subretinal fluid; ELM: external limiting membrane; EZ: ellipsoid zone; I-HRF: hyperreflective retinal foci. Post hoc multiple comparisons among clusters by Tukey–Kramer test.

## Data Availability

The original contributions presented in this study are included in the article. Further inquiries can be directed to the corresponding author.

## Abbreviations

The following abbreviations are used in this manuscript:

AI	Artificial intelligence
AMD	Age-related macular degeneration
BCVA	Best corrected visual acuity
BIC	Bayesian information criterion
CST	Central subfield thickness
DM	Diabetes mellitus
DME	Diabetic macular edema
DR	Diabetic retinopathy
ELM	External limiting membrane
ERM	Epiretinal membrane
ETDRS	Early treatment diabetic research study
EZ	Ellipsoid zone
HR	High Resolution
I-HRF	Inflammatory hyperreflective retinal foci
IRF	Intraretinal fluid
OCT	Optical coherence tomography
IQR	Interquartile range
SD	Standard deviation
SRF	Subretinal fluid
VEGF	Vascular endothelium growth factor
